# New technology must support, not restrict humaneness: a qualitative interview study on the potential influences of a new digital system on specialist palliative home care

**DOI:** 10.1186/s12904-026-02020-4

**Published:** 2026-03-04

**Authors:** Alina Weisser, Natalie Öhl, Ann-Kathrin Mahlein, Joachim Peters, Martina Simon, Stephanie Schmitt-Rüth, Anastasiya Zakreuskaya, Michael Nissen, Björn Eskofier, Christoph Ostgathe, Tobias Steigleder, Maria Heckel

**Affiliations:** 1 Department of Palliative Medicine, University Hospital Erlangen, Friedrich- Alexander-Universität Erlangen-Nürnberg (FAU), Erlangen, Germany; 2 Chair of Germanic Linguistics, Friedrich-Alexander-Universität Erlangen-Nürnberg (FAU), Erlangen, Germany; 3 Fraunhofer Center for Applied Research on Supply Chain Services SCS of the Fraunhofer Institute for Integrated Circuits IIS, Nürnberg, Germany; 4 Institute of Psychology and Behavioral Science (Faculty WEBIS), OTH Amberg- Weiden, Weiden, Germany; 5 Machine Learning and Data Analytics Lab, Friedrich-Alexander-University Erlangen- Nürnberg (FAU), Erlangen, Germany

**Keywords:** Palliative care, Home care services, Digital health technology, Telemedicine, Linguistics, Qualitative research

## Abstract

**Background:**

Electronic health applications are playing an increasingly significant role in modern healthcare systems. Therefore, the European Union proposed GAIA-X - a digital ecosystem that facilitates data exchange and sovereign data usage. TEAM-X aims to implement GAIA-X in healthcare, this study focuses on palliative care. Especially in this vulnerable context, identifying opinions of prospective users during development ensures that their needs as well as objectives and principles of palliative care are met. This study aims to gather perceptions and expectations regarding digital technologies in specialist palliative home care, exemplified by a proposed system.

**Methods:**

We conducted a qualitative study using semi-structured, problem-centered interviews with employees and informal caregivers in specialist palliative home care. Participants were introduced to the conceptual TEAM-X system as well as different care scenarios as impulses for possible changes and were asked about their expectations for benefits and challenges, and requirements for such a system. Transcripts were analyzed using content structuring qualitative analysis complemented by a linguistic analysis that examined personal attitudes towards technology through language patterns.

**Results:**

Nine employees and three informal caregivers agreed to participate. Interviewees highlighted several potential benefits of the planned system, some of them addressing challenges of the current care situation, like facilitated data exchange between healthcare professionals, reduced burden on informal caregivers, and optimized care processes. Challenges included concerns about system feasibility, data handling procedures and fears that technology might compromise humaneness. Linguistic analysis revealed varying levels of technology affinity, while the relevance for care and the alignment with palliative care principles were rated mainly high. As requirements, participants emphasized the importance of maintaining humaneness in care and ensuring that technology serves as a supportive tool rather than a restrictive element.

**Conclusions:**

Despite its conceptual nature, the TEAM-X system elicited valuable user insights, identifying both benefits and critical requirements for successful implementation. Meeting the concrete and realizable derived requirements thoroughly, such as enhancing data security, maintaining human interaction, and ensuring user-friendliness, could enable the system to support specialist palliative home care effectively. Future research should validate these findings and explore implementation in diverse healthcare contexts.

**Supplementary Information:**

The online version contains supplementary material available at 10.1186/s12904-026-02020-4.

## Background

 Electronic health is the application of technology to promote and facilitate health, well-being, and healthcare [1] and is distinguished by its interdisciplinary approach and dynamic evolution [2]. Thus, digital applications are on the rise in medicine [3]. Digital transformation has triggered changes in healthcare. One central aim is to involve patients more closely in their care, e.g., by having access to their own electronic health records (EHRs) [4].

The GAIA-X [5] initiative, driven by the European Union, too, aims to advance digitalization. It seeks to establish a trustworthy, value-based data ecosystem that facilitates data exchange and sovereign data usage. The German research project TEAM-X intends to implement those values by developing a digital ecosystem for health care based on a GAIA-X data environment. This ecosystem is designed to prioritize patients by ensuring self-determination, data sovereignty, and full control over their personal data. It further enables user-driven management and sharing of such data. Within this system, patients continuously collect and transmit it to healthcare providers. This has the potential to allow therapies and care measures (e.g., home visits) to be planned in a more targeted manner. See Supplementary Material for possible use cases of this system. At the time of this study, the system was still in development.

In Germany, palliative care needs, which encompass support for patients with life-threatening diseases and their social networks with the goal of enhancing quality of life, remain inadequately addressed [6, 7]. Furthermore, the healthcare system is burdened by excessive bureaucracy and lacks sufficient recognition of the time-intensive nature of palliative care [8]. Under this premise, one aim of TEAM-X was to develop a system to support palliative care by streamlining data exchange processes, thereby addressing existing gaps in care provision [9–11]. The system aims to adhere to the values of palliative care, with a particular focus on providing active, holistic care and enhancing the quality of life of for patients, their families and caregivers [12]. Specialist Palliative Home Care (Spezialisierte Ambulante Palliativversorgung, SAPV) is a German model of multidisciplinary palliative care delivered at home for patients with complex needs [13, 14]. This setting was chosen since digital health technologies could help bridge spatial and temporal barriers [15–18]. Previous virtual care solutions that aim to support palliative care have also predominantly focused on home-based settings [15, 19–24]. These approaches include virtual care platforms [22], the digitalization of established tools [20], and the implementation of digital applications [19].

Encouraging patients to take an active role in managing their own health is an element of providing effective care with better outcomes for patients [25, 26]. Increasing digitalization can foster patient-centeredness [26, 27]. Besides improving satisfaction and certainty, this participatory approach yields measurable improvements in clinical outcomes [4, 28–31]. It also introduces new possibilities and requirements for data provisioning, collection and analysis [3], along with challenges in designing interactions with personal data. This necessitates an interdisciplinary approach that incorporates the sensitivity with respect to this topic [2].

In addition to technical improvements, the implementation of a new digital system has the potential to change routine care practices. Therefore, ethical, legal, and social implications (ELSI) [32] must be considered, as well as user- and acceptance-related aspects that have an impact on existing care processes. Owing to the vulnerability of patients and their informal caregivers, palliative care is especially prone to complex ethical and social questions [33]. To date, little research has been conducted on the conditions under which palliative patients can benefit from a system such as TEAM-X. In particular, the perspectives of both formal (healthcare professionals) and informal caregivers have not yet been sufficiently examined [34]. Informal care involves unpaid care to dependent relatives or friends [35, 36]. There are isolated indications in literature that healthcare providers are concerned about involving palliative care patients in electronic applications [37]. Alternatively, the use of digital applications in the collection of health data can have several advantages. These include user-friendliness, immediate evaluation of data collection instruments, and increased availability of data, leading to easier decision-making [37]. Such benefits may also apply to palliative care. More positive impact on palliative care can be found in support of education, information sharing, decision-making, communication and costs [23], as well as fast and easy data exchange [38].

To ensure that the systems´ development aligns with the needs of its future users, a variety of relevant actors (e.g., healthcare professionals, patients, informal caregivers) need to be studied [34, 39–42]. Thus, the study examines expectations of healthcare professionals and informal caregivers regarding the proposed TEAM-X system. It considers anticipated benefits and risks, and how underlying individual attitudes influence these assessments. Furthermore, the manuscript examines the reasoning participants employed in their estimation processes. The study analyzed participants’ language patterns to uncover how they constructed their views about the system and what factors shaped their assessments. Based on these insights, we derive essential requirements for implementing such a system in specialist palliative home care. In summary, this paper aims to analyze perceptions and expectations regarding digital technologies in palliative care, exemplified by a proposed system. It does so by identifying the social implications of implementing a system like TEAM-X, as required for routine specialist palliative home care. This includes perspectives from healthcare professionals and informal caregivers, as well as user requirements gathered during system development.

## Methods

### Setting / Study design

Data were collected in specialist palliative home care (own home, nursing home, hospice) using problem-centered interviews [43] for content structuring qualitative analysis [44]. The linguistic conversation analysis was conducted according to the model of Liddicoat (2021), which can be assigned to the field of interactional linguistics [45].The study follows constructivist theory with a moderate constructivist position [46], influencing both the interview design and the analytical approach. The study focuses on participants’ subjective experiences and interpretations, as well as on the co-construction of meaning during interviews. It is one of two sub-studies of an interview study on the social implications of TEAM-X.

### Sampling

The sample was selected based on convenience. The inclusion and exclusion criteria can be found in Table 1. The study was aiming to recruit 6–8 healthcare professionals and informal caregivers each within the recruitment timeframe, based on the assumption of data saturation [47, 48]. Data saturation refers to the point at which no new significant themes or patterns emerged from the interviews. At this stage, the data collection was considered sufficient to address the research questions and ensure comprehensive participant characteristics were captured. The inclusion of palliative care patients was not planned since one-hour interviews were considered too burdensome. Their involvement is planned for a later stage of the system, when using a mock-up will reduce interview length and strain. All participants received a 50 € voucher for a bookstore. For brevity and clarity, all employees of specialist palliative home care teams are collectively referred to as healthcare professionals.

**Table 1:** Inclusion and exclusion criteria

	Healthcare professionals	Informal caregivers
Inclusion	Employees of a specialist palliative home care team	informal caregivers, meaning: providing unpaid care for dependent family members and friends in specialist palliative home care at least four weeks of experience
	Both: Age: min. 18 years old	
Exclusion		High emotional burden
	Both: Lack of consent to participate in the study Lack of ability to give consent Lack of language skills	

### Recruitment

Recruitment took place between January and May 2023. Healthcare professionals were recruited through various channels: specialist palliative home care teams previously collaborating with the study’s department, the German Association for Palliative Medicine (DGP), a congress of the DGP (WAT), the Association for Specialist Palliative Home Care in Bavaria, and via social media (Instagram). Recruitment sources for informal caregivers included hospice associations previously collaborating with the study’s department, the patient and public involvement initiative (PPI) of the study’s department, social media (Instagram), and snowballing.

### Data collection

Interviews were audio-recorded via Zoom Video Communications with no presence of non-participants. A.W. (psychologist, research assistant) conducted the interviews in German following specialized interview training. Participants were unknown to the researcher prior to data collection. Slides illustrating potential TEAM-X ecosystem setups and care scenarios were presented to stimulate discussion (for a shortened version, see supplementary material). The scenarios depicted “recognition of palliative care needs”, “monitoring of symptom burden”, “monitoring of health status” or “opioid prescription” and “advance care planning”. In line with the principles of problem-centered interviews [43], the scenarios served as stimuli to openly explore participants’ subjective perceptions to palliative care practice, data exchange processes and changes through TEAM-X. This approach combined open-ended questioning with focused follow-ups, aiming to gain in-depth insights into individual orientations and practices.

The interview guide (for a shortened version, see supplementary material) was informed by the CARE-HOUSE Framework [49]. The CARE-HOUSE Framework conceptualizes social implications of digital health technologies in palliative care. It suggests that social implications may manifest in the characteristics and roles of actors, their interactions, tasks and processes, as well as the context in which they are embedded. The interview guide included questions about data exchange practices in routine palliative care practice, and the potential impact of introducing a new digital patient centered technology for data exchange. Participants’ viewpoints and concerns regarding this technological innovation were also included. The interviewees were encouraged to provide their candid opinions on the proposed system and were explicitly invited to share any criticisms without reservation. Reflexive fieldnotes were made after the interviews. The local ethics review board approved the study on January 9, 2023 (22-407-B).

### Data analysis

Transcription was conducted following Pehl and Dresing [50] were analyzed according to structured content analysis [46] with MAXQDA 2022 / 2024 (release 22.0.7/ 24.0.0) [51]. For quality assurance purposes, A.W. and A.K.M (master’s student) carried out separate coding. They checked for intercoder agreement regularly, resolving disagreements through discussion and leading to final decisions. Difficult passages were discussed with an additional research assistant in feedback loops. After each iteration, the coding of the previous interviews was adjusted. At the 6-interview mark (55%), a third-party researcher (M.H., social worker and nursing scientist) conducted a consistency check to verify the consistency and degree of differentiation between categories. Throughout the process, the MAXQDA logbook feature and memos were used to record established coding rules, modifications to the code system, and decisions regarding subsequent coding steps. Additionally, a linguistic analysis was conducted to complement the content analysis to gain insights on the participant’s implicit perceptions of technology and attitudes due to the analysis of how participants speak about it (see chapter linguistic analysis).

### Coding system

The deductive content coding system was built upon the research questions, interview guide, and components of the CARE-HOUSE Framework [49], . The inductive subcategories were subsequently built during the coding process (see Table 2). The concept of humaneness was employed based on Max Black’s (1979) understanding of humane interaction as involving attentiveness, consideration, and mutuality of interrelationship [52].

**Table 2:** Content coding system

**assessment of the current situation**	functioning processes	treatment of patients
		communication, data collection and exchange
		processes^1^
	difficulties and challenges	treatment of patients
		communication, data collection and exchange
		Not (sufficiently) functioning care processes^1^
		informal caregivers are overburdened^2^
	**general wishes**	
**assessment of the prospective situation with the technology**	**relevance**	
	**benefits**	**general improvement to palliative care practices**
		**improvement of care**
		**self-determination and safety**
		**optimized assessment of health status**
		**optimization of processes**
		**relief for the users** ^**2**^
	**risks and concerns**	**feasibility**
		**data handling processes** **(data security, protection and transfer)**
		**humaneness**
		**general concerns**
		**self-determination** ^**2**^
	**willingness to use the new technology**	
**requirements**	**exclusion criteria**	
	**requirements**	**functional**
		**conceptual**
		**person-related**

Legend: ^1^: only named by healthcare professionals; ^2^: only named by informal caregivers; bold: relevant for the current study

Formal codes contributed to differentiating between text passages referring to the current and prospective situations. Only the prospective situation was used for analysis. Formal codes also marked passages in which interviewees referred to one of the proposed scenarios (see the data analysis section). Additionally, codes highlighted paragraphs in which interviewees described expectations for good palliative care.

### Linguistic analysis

Linguistic aspects were included in the analysis to identify perceptions of technology and attitudes, especially the affinity for digitalization. It is useful to describe linguistic phenomena beneath the surface structure of language, because the form of what is said allows precise statements to be made about the attitudes and values of the communicators that are not explicitly expressed [53]. In parallel with the other analysis steps, two members of the linguistic department (J.P., researcher; S.F., researcher) conducted an independent linguistic analysis of the data sets to ensure inter-coder reliability. The relevant phenomena of a textual linguistic description were annotated in MAXQDA 2024 (release 24.0.0) [51]. The following linguistic categories were systematically examined: sentiment and emotion words, pronoun use, use of technical language, metaphors and metonymies. Sentence structure and complexity, paraphrases, use of adverbs and adjectives, word formation patterns as well as recurring topics or statements were analyzed [53, 54]. The linguistic method of agency analysis examines power dynamics by identifying entities that frequently appear as active agents in texts, whether through direct actions or metaphorical representation [55, 56]. This reveals the distribution of power in relationships between actors (such as healthcare providers and digital tools). It shows who is portrayed as having independent agency versus being controlled by others [57, 58]. Based on the parameters examined, the subjects were divided into three different categories of technology affinity: high, medium and low affinity. The factors in the analysis included the use of technical language and technical group language, entity conceptualization, and pronoun use. Expressed subjective attitudes and emotions regarding the use of digital technology (digital-related sentiment) were also considered. The criteria are defined in Table 3. At the end, we compared the results of the linguistic analysis with the other analysis steps, identifying similarities and differences.

**Table 3:** Classification criteria regarding affinity with technology

high affinity	low affinity
- conceptualizes entities with concrete nouns - technical language and terminology are mastered and used competently - the person likes to talk about digitization - positive sentiment, the topic is positively evaluated with emotion words - no uncertainties are highlighted by the speaker	- conceptualizes entities with paraphrases, pronouns or metaphors - technical language and terminology are replaced by words with general semantics - the topic of digitalization is structurally avoided - negative sentiment, the topic is negatively evaluated with emotion words - uncertainties are strongly marked in language by verba sentiendi, adverbs or conjunctives (“it could be that …”/“I believe …”/“possibly”, “perhaps”)

Legend: The classification was defined as a continuum. The participants who could not be classified as “high” or “low” affinity, were assigned a “medium”

### Use of generative AI and AI-assisted technologies in the writing process

During the preparation of this work the authors used ChatGPT, DeepL and SpringerNature’s language editing in order to correct language in the writing process and improve readability and language of this manuscript. After using this tool/service, the author reviewed and edited the content as needed and takes full responsibility for the content of the published article.

## Results

This section presents the study results. Following recruitment and participant characteristics, participants’ general perceptions of palliative care are summarized to contextualize their evaluation of the system. Anticipated benefits, risks, and concerns regarding implementation are then outlined. This is followed by an analysis of the system’s perceived relevance and alignment with palliative care principles, as can be derived from content analysis. Based on the linguistic analysis, the findings are further examined in relation to participants’ technological affinity. This aims to explore its potential influence on how they evaluate and interpret the system. Finally, factors potentially influencing acceptance and user-defined requirements for implementation are discussed.

### Recruitment

Of eight healthcare professionals who reported back, all were eligible. Among four informal caregivers reporting back to recruitment, one informal caregiver was excluded because of a one-week period of home care, which was considered too short. The reasons for refusal were not systematically assessed. Healthcare professionals most frequently expressed a lack of time or interest. It turned out to be difficult to reach informal caregivers with the required experience and who were not too emotionally burdened. Despite aiming to recruit 6–8 informal caregivers, only three were reached within the recruitment time frame. Given the importance and value of the informal caregivers’ perspective, it was decided to include the collected data as supplementary information. However, a direct comparison was not feasible. All participants completed the study, and no interviews required repetition.

### Participant characteristics

Eleven interviews occurred between March and May 2023 until data saturation was reached for healthcare professionals. The average duration of the interviews was 54:46 min (Standard Deviation 7.55). Two of the participants were male, and the other nine were female. The average age of the participants was 52 years (SD = 10.4; min = 34; max = 68). More details about the interviewees can be found at Table 4. To protect participant anonymity, age was reported in categorized age ranges.

**Table 4:** Demographics of healthcare professionals and informal caregivers

healthcare professionals							
participant	age range	gender		profession		years of experience in palliative care	
2	[65–70]	male		medical director		eleven or more years	
3	[55–60]	female		office management^*^		eleven or more years	
4	[55–60]	female		medical director		eleven or more years	
5	[45–50]	female		nurse		six to ten years	
6	[45–50]	female		nursing director		eleven or more years	
7	[55–60]	female		nurse		eleven or more years	
9	[45–50]	male		team leader		eleven or more years	
10	[30–35]	female		physician		less than one year	

**informal caregiver**							
participant	age range	gender	relation to patient		distribution of responsibility		duration of care
1	[40–45]	female	granddaughter		team of three		two and a half years
8	[60–65]	female	partner		primarily responsible		two and a half months
11	[65–70]	female	daughter		primarily responsible		one and a half years

Legend: ^*^: This participant was the office manager of a specialist palliative home care. In the following, she will be included in the term “healthcare professionals”

### Participants’ definition and perception of palliative care

Palliative care as “*accompaniment to life”* (Int. 7, healthcare professional) sums up the healthcare professionals’ perceptions, involving a whole-person and social systems approach, reducing symptom burden and providing stability and security through time and personal connection and contact. Concerning the treatment of patients in the current situation, they valued the possibility of maintaining good palliative care, having enough time for the patients, the ability to act comprehensively in response to the patients’ needs, having good documentation and having a special relationship with the patients with personal contact as a strength.

Informal caregivers perceived the consistency of support, respect and realization of patients’ will, wishes and rights as good palliative care. They focused more on the care structure, naming good (specialized palliative) outpatient care and support by volunteers.

### Aspects of implementation of the system

#### Anticipated benefits

The interviewees described data exchange in the current situation as seemingly quite complex, time-consuming and extensive. They named communication barriers, insufficient interprofessional communication, stemming from various sources such as a lack of centralized data storage or limited access to information from other institutions.

“*Because then the communication just didn’t work at all anymore*,* and*,* I think*,* for that*,* it would be great*,* because then it goes through five corners. […] But the others don’t communicate with each other.“* Int. 1, informal caregiver; *„If you*,* I don’t know*,* were to call*,* let’s say*,* the department A and say*,* ‘Hey*,* I’d like to talk to the treating physician*,*’ it’s difficult*,* right? So I think the networking there is not as natural as it is with the palliative care unit. (…) Whether that can even be achieved or expected in such a large institution is questionable. But I think it would be nice if there were more data exchange.“*.

Int. 5, healthcare professional.

Healthcare professionals criticized data management processes such as excessive reliance on paper-based systems leading to high documentation efforts or a lack of structured processes.

“*Or I would wish that it were more structured. Some things can be structured*,* you know. So*,* despite all the individuality in personal care*,* it’s certainly possible to distill some structure. And that would definitely be helpful at times*.“.

Int. 5, healthcare professional.

Several institutional barriers, such as data privacy measures hindering care processes, and various institutions resisting digitalization efforts, have been identified by healthcare professionals.

“*Most teams with ISPC work with iPads […]. This has always failed for us due to data protection issues*,* because it wasn’t considered good enough by our IT department. I do see that it’s a bit difficult and could be easier if it were legally compliant with data protection regulations*,* to the point where even our IT department would say*,* ‘Okay*,* this works*‘“.

Int. 9, healthcare professional.

The healthcare system in general was described as “*regressive”* (Int. 10, healthcare professional) and “*increasing difficulties in providing care*” (Int. 5, healthcare professional) were expected in the future. Informal caregivers described themselves in a moderating role, informing healthcare professionals about changes in patients’ health status, ensuring the implementation of care plans and taking a responsible role for data exchange and interprofessional communication.

“*If I remember*,* I was insanely busy with it. That’s right*,* I also documented it*,* I’m only now realising that. […] I was also really busy with this planning and updating of ‘What do we have to give now*,* what else can we do now?’ and then rushing to the pharmacy again […]. But I was already very involved in this medication*,* exactly*,* administration*,* documentation and then adjustment again.”* Int. 8, informal caregiver.

Some of the identified challenges could be addressed by the new digital system. The interviewees emphasized that the two main functions of the proposed system (centralized data storage and easier data exchange) could foster data exchange, communication and cooperation between actors, prevent data loss and reduce moderating tasks for informal caregivers.

“*If we just knew*,* if we could log in and know ‘this is how it is*,*’ that would be great. I would really benefit from that. We also care for the hospice*,* so if I had that information without having to bother anyone*,* that would be really valuable for me. Those would be the two points of contact I find most important. And the oncologists*,* if the patients are still being treated there*.“.

Int. 4, healthcare professional.

Additionally, both groups assumed the TEAM-X system to be a general improvement to palliative care practices.

*I believe that this could improve EVERYTHING.*

Int. 1, informal caregiver.

Additionally, continuous assessment of symptoms, having access to data from everywhere or simplified prescription ordering at the pharmacy was perceived as beneficial by healthcare professionals.

“*So*,* the symptom burden*,* opioid management*,* and this simplified data exchange – brilliant*.“.

Int. 7, healthcare professional.

Having access to information prior to visits could optimize treatment.

*Which of course would perhaps simplify some things*,* because*,* as I’ve already said*,* with some patients we’re just getting there and don’t have any documents yet*,* so if I could see them in advance*,* it would probably make things easier.*

Int 10, healthcare professional.

Informal caregivers described possible benefits for the care of patients speaking other languages. Both groups identified potential time savings that they wished to be allocated to humaneness and personal contact.

*If these objective parameters are already recorded via this system*,* so to speak*,* then there is simply more time for discussions about the remaining time of life*,* about approaching death*,* what can I still do for myself*,* what can I still do for my companion?*

Int. 8, healthcare professional.

Both groups wished support of actors’ self-determination, and healthcare professionals stressed creating a feeling of responsibility for informal caregivers, thus feeling important and needed.

*[…] and I think there’s definitely an advantage for the relatives*,* who would still have a sense of responsibility*,* right?*

Int. 7, healthcare professional.

Assuring patients will receive help when required can support their need for safety (healthcare professionals). Informal caregivers observed a relief for all users resulting from less documentation effort, fewer mediating tasks and organizational tasks for informal caregivers and better involvement.

*And that’s what I would hope for*,* that it would be easy /. That I’m part of the whole thing and I can still see what’s happening with the nurse or the dying person*,* but I can see that I’m more or less just a spectator and the others are communicating and exchanging ideas and getting the best possible care for the dying person*.

Int. 1, informal caregiver.

The interviewees suggested potential shifts in tasks and roles with the introduction of the new system. Healthcare professionals might take on more educational roles in managing the new system, whereas informal caregivers might adopt a more passive stance. All the interviewees stressed the continued necessity of personal contact in everyday care, particularly for sensitive aspects such as advance care planning, for which the system should not interfere.

“*I also like the digital education about the advance care planning. You can read about it*,* you can listen to it. But a personal conversation*,* what are my wishes and needs? That can never replace a personal conversation.“*.

Int. 3, healthcare professional;

*This reaction to the blink of an eye*,* to a (…) moan*,* breathing*,* I don’t know what can perhaps not be recognised. But what an experienced palliative care doctor can recognise. And that’s what makes the difference. (…) And that works with such aids*,* which must always*,* always be subordinate to the person*,* in my opinion.*

Int.8, informal caregiver.

#### Risks and concerns of prospective users

Healthcare professionals expressed concerns that the image of a transparent patient could be threatening and that informal caregivers feared the system not being suitable for care at the end of life.

Well, I could just imagine, maybe, if it really comes to the end and you realize that it is all about hours or minutes or something, that you don’t necessarily need to access something digital, but that it’s just the human element that counts, the personal aspect. So, really, when you’re in a situation like this, you don’t try to exchange contacts or information or anything else.

Int. 11, informal caregiver.

Regarding feasibility, healthcare professionals raised concerns that the system could lead to additional burdens, for healthcare professionals (e.g., constant availability), patients (e.g., unease about monitoring) and informal caregivers (e.g., overburden due to too many functions).

*That this is simply an additional task for them to say ‘I still have to remember*,* I have to maintain this system*,* I have to pull up every doctor’s letter. I have to release data so that the other person can access it. […] The idea of doing that for eighty or eighty-five patients who die relatively quickly […] is almost unimaginable for me*.

Int. 3, healthcare professional.

Organisational (e.g., that palliative care guidelines could stand in the way of digitalization of opioid management) and technical feasibility (e.g., wireless internet connection availability) were issues raised by healthcare professionals.

“*Digital opioid managers*,* especially for recording side effects in home based specialist palliative care*,* are counterproductive because opioids are generally not given according to the guidelines*,* but according to their effect*.“.

Int. 2, healthcare professional.

Informal caregivers named personal attitudes (e.g., negative connotations to be monitored) and lacking competencies or capabilities (e.g., not having the technical competencies to use the app, not having the cognitive means or vigilance to use the app) as possible hindrances for implementation, suggesting implementation might only be possible with fit/certain patients.

Both saw risks in data handling procedures (security, protection, entry and exchange). Informal caregivers focused on data security and protection. Healthcare professionals described detailed aspects such as handling with data sovereignty, data protection, loss of data quality (e.g., data being one-dimensional without personal questions), data overload or loss of control by automated data transfer.

“*On the other hand*,* if this happens automatically*,* I no longer have an overview*,* so to speak - is that really what the others need? […] I can no longer do anything myself*,* so to speak*,* to forward information that I now consider particularly noteworthy […].“*.

Int. 6, healthcare professional.

All interviewees expressed concerns that technology might fail to adequately reflect reality, emphasizing that it would struggle to fully grasp the complexity and diversity of care situations, thereby potentially compromising humaneness.

*Unfortunately*,* this does not reflect palliative care perception electronically or digitally.*

Int. 2, healthcare professional.

Healthcare professionals anticipated that the role of informal caregivers might change and that the holism of a human cannot be assessed. They raised concerns about decisions made by a machine. Informal caregivers feared that the self-determination of patients would be threatened by substitute data entry or if different informal caregivers had access to and power over data while not agreeing about the treatment.

#### Perceived relevance and alignment with the palliative care context

Seven out of eight healthcare professionals rated the system as being moderately to highly relevant to palliative care (see Table 5). In the specialist palliative home care setting, the participants did not perceive the digital palliative needs assessment scenario as immediately relevant. They recognized its potential value earlier in the patient journey, before outpatient care involvement. They considered the system useful for patients living alone (e.g., monitoring their health status). The relevance of all functions of the system were questioned (all scenarios) except facilitated data exchange, partially because they were rated as not meaningful, not relevant, set too early in the patient journey or not practicable. Informal caregivers rated the relevance and meaningfulness of the system as high and thought it could also be helpful in other areas of application.

**Table 5:** Comparison of participants’ affinity for digitalization (linguistic classification), assessment of relevance and alignment with palliative care principles

interviewee, age and position	affinity for technology	assessment of relevance	alignment with palliative care principles
healthcare professionals			
2	**high**	**moderate** relevance lies in simultaneous access to data	**no** concerns about how patients and informal caregivers handle knowledge; concerns that patients and informal caregivers don’t know how to handle data sovereignty
3	**low**	**low** cannot imagine it working: patients will not agree and won’t be able to operate it; sees access of all actors as an obstacle	**no** personal contact is important; no more tasks for patients and informal caregivers; not realizable due to too many patients
4	**high**	**high** better planning of visiting routes	**yes** since constant contact is possible; possibility of reacting fast; no disadvantages
5	**high**	**growing** due to difficulties in the care situation	**yes** if personal contact is priority
6	**medium**	**high** since digitalization is inhibitable	**yes** by structured monitoring; concerns about restricted personal contact
7	**medium**	**high** especially facilitated data exchange, digital monitoring of symptom burden and opioid management	**yes** but danger of restlessness of patients because of constant knowledge about own health status
9	**high**	**moderate** only facilitated data exchange is relevant all other functions are not	*no clear statement* it shouldn’t be in the way of personal contact; good considerations about functions needed interviewee had frustrating experiences with new technical systems
10	**medium**	**high** especially facilitated data exchange and centralized data location	**yes** if personal contact is not used for explaining the system good: more information in advance for healthcare professionals and for patients/informal caregivers
informal caregivers			
1	**medium**	**high** best as subfunction of health insurance app	**yes** it can only get better
8	**high**	**high** especially facilitated data exchange	**yes** if there is more time for personal contact
11	**medium**	**high**	**yes** if there is more time for more important things than data exchange

Legend: classifications are highlighted in bold to improve readability

Among healthcare professionals, five respondents rated the system as being aligned with palliative care principles, two rated it inappropriate and one did not provide a clear statement (Table 5). Among informal caregivers, all interviewees rated the system as aligning with palliative care principles and expected major improvement in the current insufficient situation, given that the system enabled more time for personal contact and humanity.

#### Individual attitudes and the influence of technology affinity

The comparison of participants’ statements regarding relevance and alignment with palliative principles to the linguistic classification of their technology affinity (Table 5) suggests that technology affinity may influence the perceived relevance of the system. Linguistic analysis revealed that some participants showed avoidance strategies and did not want to conceptualize entities using the respective technical terms. They replaced them with pronouns or words with more general semantics (“it”/“the system”/“such a system”) and were deemed to be less technically savvy. The use of technical language and emotional words to illustrate their positive attitudes indicates higher digital competence among interviewees. In addition, people with less technical affinity tended to present difficult issues in a simplified manner or even describe them metaphorically.

Healthcare professionals assessed relevance and alignment with palliative care principles very differently and were able to report their opinions in a differentiated way. No trends were found concerning the age of the participants. Notably, the only person to rate relevance and alignment with palliative care principles as low was not working as a healthcare professional but in office administration. All informal caregivers found the relevance and alignment with palliative care principles to be high, while having medium to high affinity for technology. They reported their opinions in a more general, less differentiated manner than healthcare professionals did.

#### Anticipated aspects to influence acceptance of the system

The participants identified several factors that the acceptance of the new system was impacted by several factors. Healthcare professionals referred to decisive factors such as health status, age, other demographic aspects or technological affinity of the user. Both groups assessed younger people to be more likely to use it as they have a more positive view of technology.

“*Some yes*,* some not. Yes. Especially the younger ones – I’m totally convinced that they’re really into it*,* for sure*,* right?*“.

Int 7. Healthcare professional.

People living in rural regions may have difficulties accessing the internet, others may have no available devices or restricted financial resources.

Both groups named exclusion criteria for implementation. Healthcare professionals pointed to factors such as a lack of motivation, insufficient cognitive ability, significant physical weakness, or an inability to operate a smartphone. They believed that many of those criteria are applicable to the palliative care population. Informal caregivers, on the other hand, defined exclusion criteria that were more technology-dependent, i.e., systems with overly complex handling or excessive time demands. Addressing these issues will be essential when the new system is implemented.

### Requirements for the system

To ensure that the benefits of the new system can reach their full potential, manifold requirements must be met to counteract the identified challenges. Table 6 gives an overview of the requirements (functional and non-functional [59, 60]) derived from the interviews.

**Table 6:** Functional and non-functional requirements

category	requirements
functional requirements	
handling and presentation of data	data security^1,2,4^
	centralized data location^3^
	system based predefinition of input and output data (e.g., limitation of amount) ^1,2,4^
	structural overview over patient data^1,2^
	printable data^1^
	verbal data entry^1,2^
underlying structure	well-sustained underlying infrastructure^1^
	facilitated data exchange between all actors^1,3^
	compatibility (with existing systems, e.g., ispc; subfunction of health insurance app; with all operating systems)^1^
	depiction of heterogenous social/organizational systems^1,4^
	self-determined sharing of data (each actor can be added actively)^1,2,4^
provision of technical possibilities of functions	provision of information material (e.g., as explanatory video)^1^
	substitute data entry (healthcare professionals or informal caregivers)^1^
	supervision of several persons (by informal caregivers)^1^
	communication tool^2^
	solution for technical failure^1,2,4^
user friendliness	User-friendliness in general^1,2,4^
	intuitive layout^1^
	low threshold^1^
non-functional requirements	
conceptual requirements	
balance between technology and the human element	human control: decisions should be made by hcp, not the system^1,2,4^
	balanced ratio between information from the system and personal contact^1,2,4^
	good reflection on which tasks should be taken on by the system^1,2,4^
consideration of technological solutions	clarification of handling with inactive accounts/deceased patients^1^
	good consideration of concept of substitute data entry^2^
consideration of organizational aspects (care practice)	consideration of impact of technology on care practice^1,4^
	consideration of palliative guidelines^3^
	definition of responsibilities (for education of healthcare professionals, for support of informal caregivers and patients or for data interpretation) ^1,2^
	necessity of on-call service for monitoring of symptom burden^1,4^
	timely scheduling of notifications for the completion of advanced care planning^1^
	adaptation to increased number of palliative patients^1,4^
	telephone consultations between healthcare professionals should persist^1,4^
	consideration of options when health status is low^2^
	good integration and discharge of informal caregivers^1,2,4^
consideration of organizational aspects (general)	good organizational structure^1,4^
	good depiction of reality^2^
	legal protection^1,4^
	little expenditure for all actors (e.g., time, documentation)^1,2,4^
	clarification of cooperation with health insurances^1^
	clarification of financing^1^
	alternatives for people who don’t want or can’t to use the system^1,2^
	successful introduction of the system only if majority of users is interested and convinced ^1,4^
	consideration of handling with reservations and fears of users^2^
	clarification if informal caregivers need to install the app, too^1^
person-related requirements	
person-related requirements in general	humaneness/ personal contact should stay in the foreground^1,2,4^
	consideration of the need of human support^1,2^
	patients have to be aware that a person will get in contact with them, additionally to giving the data to the system^1^
	self-determined and autonomous use of the system (patients)^1,3,4^
	ensuring benefits for the dying^1,4^
requirements concerning access to the system	fast and easy access^1,2^
	free access^1^
	access to technological devices^1,2^
	access to internet^2^
	location-independent access^1^
	preselection of people with access^1^
	actor-specific access^1^
requirements concerning education and involvement	necessity of well-trained nurses and informal caregivers that know patient well^1,2,4^
	usage of the information videos to educate about the system^1^
	patients need to be familiar with the usage of new technology^1,4^

Legend: ^1^: stated directly; ^2^: derived from risks and concerns; ^3^: derived from general wishes; ^4^: derived from linguistic analysis

*hcp* healthcare professional

## Discussion

This study examines how healthcare professionals and informal caregivers perceive the new digital system and the changes they expect from its implementation. It also explores the functional and non-functional requirements they determine for the system to effectively support palliative care delivery in home care. Although the results were generated in the context of the TEAM-X system, they are likely transferable to similar emerging digital technologies in palliative care.

### Main results

The perspectives of the interviewees on a new digital system such as TEAM-X are multifaceted, encompassing a range of perspectives and considerations. A comprehensive review of the statements reveals numerous arguments that must be considered before implementing such a system.

Participants expressed dissatisfaction with various aspects of the current situation, including a lack of structure and overly complex processes. They noted that these issues led to difficulties in data exchange and placed a burdensome coordinating role on informal caregivers. A centralized digital system was perceived as a potential solution to improve coordination, reduce workload, and provide support, without replacing personal care, while potentially relieving informal caregivers. However, both groups raised concerns regarding the system’s feasibility and data handling procedures, particularly with respect to data security. They feared that such a system might fail to adequately capture the human aspects of end-of-life care. The majority of interviewees considered the system to align with palliative care principles. Most rated it as at least moderately relevant for palliative care settings. Linguistic analysis supported the impression from content structuring that general attitudes toward technology may underlie participants’ responses, an aspect not fully accessible through content analysis alone. It provided valuable insights into how such attitudes may influence perceptions and evaluation of the system.

The functional, conceptual and person-related requirements were stated directly and indirectly. These could be derived through the analysis of concerns, the general wishes of participants and the linguistic analysis. The participants emphasized the importance of maintaining personal contact, suggesting that technology should serve to reinforce, rather than impede, the human element. These statements are in line with previous studies on health care professionals’ perspectives, experiences and preferences toward digital technology use in palliative care in Germany [34]. They also correspond with research on actors’ perspectives on virtual care in palliative care in Canada [22], and on patients’ trust in AI and therapeutic decisions made by AI for prostate cancer patients in Germany [61].

Overall, the participants concluded that the system could have significant advantages for palliative care, given that the elaborate requirements were met. They stated that fast and easy data exchange was the most important benefit of the system, given that the underlying structure allows for fast and reliable reactions. Fast and easy data exchange could already be detected as a benefit of digital health applications before. This includes a systematic meta-review on digital health interventions in palliative care and a study on technology acceptance in palliative care in Canada [23, 38].

For this system to reach its full potential, users must be both able and willing to engage with it. The interviewees indicated that demographic factors and technological affinity influence system adoption. This aligns with the literature showing that older people have less technical affinity [62]. Both demographics [63] and technical affinity [61] were found to affect access to and trust in digital health technologies among vulnerable patients. The participants’ statements concerning impaired cognition and weakness hindering patients from using the technology could further challenge the implementation of new digital technologies. However, further studies are needed to analyze this tendency in depth.

Given the mixed affinity for technology and varying levels of comfort with digital systems, we suggest developing targeted training courses for future users. These could enhance technological expertise and help users overcome their fears or reservations about the system. Further research could explore whether some roles within specialist palliative home care teams tend to be more or less skeptical of new technologies. This could help identify a targeted way of support. As suggested by participants, staff with high technical affinity could act as points of contact and support for their colleagues. Such training could possibly counteract some people’s reservations about both technology and palliative care in general.

All other functions of the system were questioned to some extent by the participants. In particular, the digital implementation of advance care planning was considered not useful. This is especially interesting since there are tools that have this function and a digital approach is recommended by other studies [64].

Recommendations for technical implementation can be derived from the requirements. For instance, the study indicates that informal caregivers often take on organizational tasks, increasing their workload. Consequently, the system should be designed in a way that reduces these administrative tasks. Automated notifications or integrated communication channels could help streamline organizational processes and relieve informal caregivers. Previous studies show that healthcare professionals spend nearly two hours on electronic health records and desk work for every hour of direct patient care [65]. Reducing administrative and documentation tasks could therefore also significantly benefit healthcare professionals. It is evident from the study that all the actors may have different requirements and expectations of the system. A modular and customizable design should be considered with functions such as flexible notification options or a simple selection of data protection settings.

### Strengths and limitations of the study

This study is one of the few to focus on how healthcare professionals and informal caregivers perceive the implementations new digital technologies in specialist palliative home care. The strengths of this study lie in its comprehensive data collection, targeted expertise, and systematic analysis. These aspects make it a valuable contribution to understanding digitalization in palliative home care. Conducting the study within the context of specialist palliative home care ensures that the findings are directly relevant to this specific field. The inclusion of participants with diverse criteria in terms of age, gender, and relationship with patients enriched the study. This diversity enabled a well-rounded understanding of the topic.

The chosen method proved valuable in gathering the reflections of the interviewees, encompassing both their professional and personal perspectives. The interview method provided the flexibility needed to explore the topic, allowing participants to express their experiences and perspectives in depth. This was particularly helpful given the challenges of the study setup. The system was still under development and could only be presented conceptually and in its basic features. TEAM-X had to be presented prospectively on the basis of use cases and the interviewees had to imagine it.

It is conceivable that a selection bias may have occurred, favoring individuals with a general interest in digitalization. However, an analysis of the interview responses suggests a broad range of perspectives was captured. Some participants explicitly expressed a desire to share critical opinions about digitalization.

Although systematic, content structuring qualitative analysis may reduce the richness of qualitative data. This is because it focuses mainly on predefined categories derived from the CARE-HOUSE Framework [49]. This could have resulted in the loss of some nuanced insights that fall outside of the framework categories. However, this was captured through inductive subcodes.

Recruiting participants proved to be a significant challenge, primarily due to the limited availability of healthcare professionals and the difficulties in reaching informal caregivers. Some healthcare professionals conducted the interviews at work or under time constraints. Only three informal caregivers could be included due to limited availability. As a result, the sample size is relatively small. Thus, the experiences and perspectives captured may not fully represent the diversity of all individuals involved. The predominance of healthcare professionals with high expertise might have skewed the results toward professional perspectives. Furthermore, data saturation regarding the perspectives of informal caregivers may be limited due to the small sample size. Nevertheless, their contributions were considered valuable and were included to provide a more comprehensive understanding of palliative care practice. In contrast, data saturation for healthcare professionals was carefully monitored, and recruitment was concluded once thematic repetition occurred. The insights provided by informal caregivers in this study may serve as a foundation for future research aiming to explore their perspectives more thoroughly.

A key limitation of this study is the absence of patient perspectives. The inclusion of palliative care patients was not planned at this stage to ensure the lowest possible burden (see sampling section). Future studies should therefore include patient perspectives to ensure a more comprehensive understanding of user needs.

Since conducting an interview requires that both participants influence the interview situation, it should be disclosed that the researcher was a psychologist which might have influenced the course of the interview. Her quantitative background could also have impacted the presentation of the results. As with any semi-structured interview, there is a risk that the interviewer’s questions, tone, or follow-up prompts could inadvertently influence participants’ responses, introducing bias into the data collection process. This possible impact has been counteracted by conducting an interview training to foster open questions and emphasizing at the beginning of the interviews that the interviewees should not hold back and express their honest opinions and criticism.

### What this study adds

Early assessment of the influences of new digital technologies can support care, minimize risks, promote user-centered design, and ensure alignment with the principles of palliative care. This work highlights key aspects to consider before implementing new digital technologies in palliative care. It identifies important aspects that need to be investigated further. These include the influence of demographics and technology affinity on patients’ readiness to use new technologies. Further studies could develop guidelines that demonstrate how technologies can complement human interactions without replacing them. They should also ensure that the time saved by the system is actually reinvested in personal contact.

## Conclusion

The estimates of possible changes in palliative care practice due to new technologies vary significantly, and their requirements are manifold. When interpreting the data and implementing such systems in this field, it is imperative to pay close attention to the human element. Among the priorities are maintaining personal contact, using technology as a supportive tool, and addressing users’ negative experiences, personal fears and reservations. Certain functions of the proposed new system, such as facilitating data exchange, could support specialist palliative home care. This may lead to enhanced communication and more efficient discharge of all actors. However, future research should further include the patients’ perspective, validate these findings and explore implementation in diverse healthcare contexts.

## Supplementary Information

Supplementary Material 1: Shortened presentation of the TEAM-X system

Supplementary Material 2: Shortened interview guide

## Data Availability

The datasets generated and analyzed during the current study are not publicly available since data protection information to participants guaranteed their anonymity in any presentation or dissemination of the study’s findings. As the participants´ responses partly contained detailed information on the specialist palliative home care teams, it is not possible to anonymise data completely. The raw data is therefore unavailable. The datasets are available from the corresponding author on reasonable request.
