# Supplementary material for: New technology must support, not restrict humaneness: a qualitative interview study on the potential influences of a new digital system on specialist palliative home care
Source: BMC Palliat Care. 2026 Mar 4;25:87. doi: 10.1186/s12904-026-02020-4 (PMC13063643; doi:10.1186/s12904-026-02020-4)

How is data currently recorded and passed on in everyday SAPV care?

TEAM-X
Trusted Ecosystem of Applied Medical Data eXchange

Example: typical patient

- monitoring of symptom burden
- opioid prescription
- Other scenarios
- recognition of palliative care needs
- monitoring of health status
- advance care planning

Care Situation with the TEAM-X-system
Imagine the system was installed tomorrow - what would change?

Mrs Meier
68 years

Digital monitoring of symptom burden

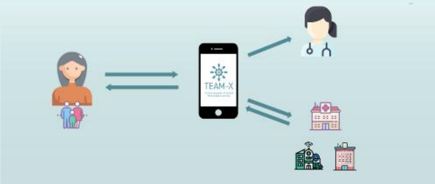

Digital opioid prescription and recording of (side) effects

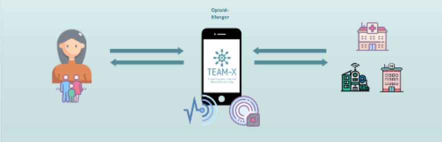

Simplified data exchange between practitioners

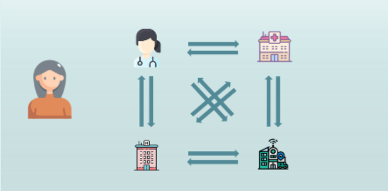

Digital recognition of palliative care needs

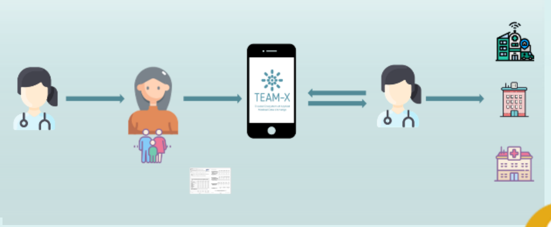

Digital monitoring of health status with the integration of biometric data

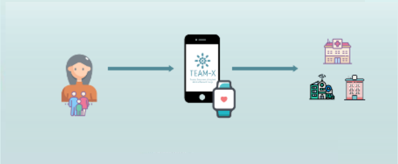

Digital information about advance care planning

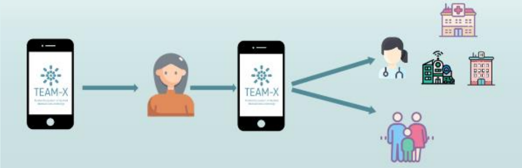

General Functions of TEAM-X

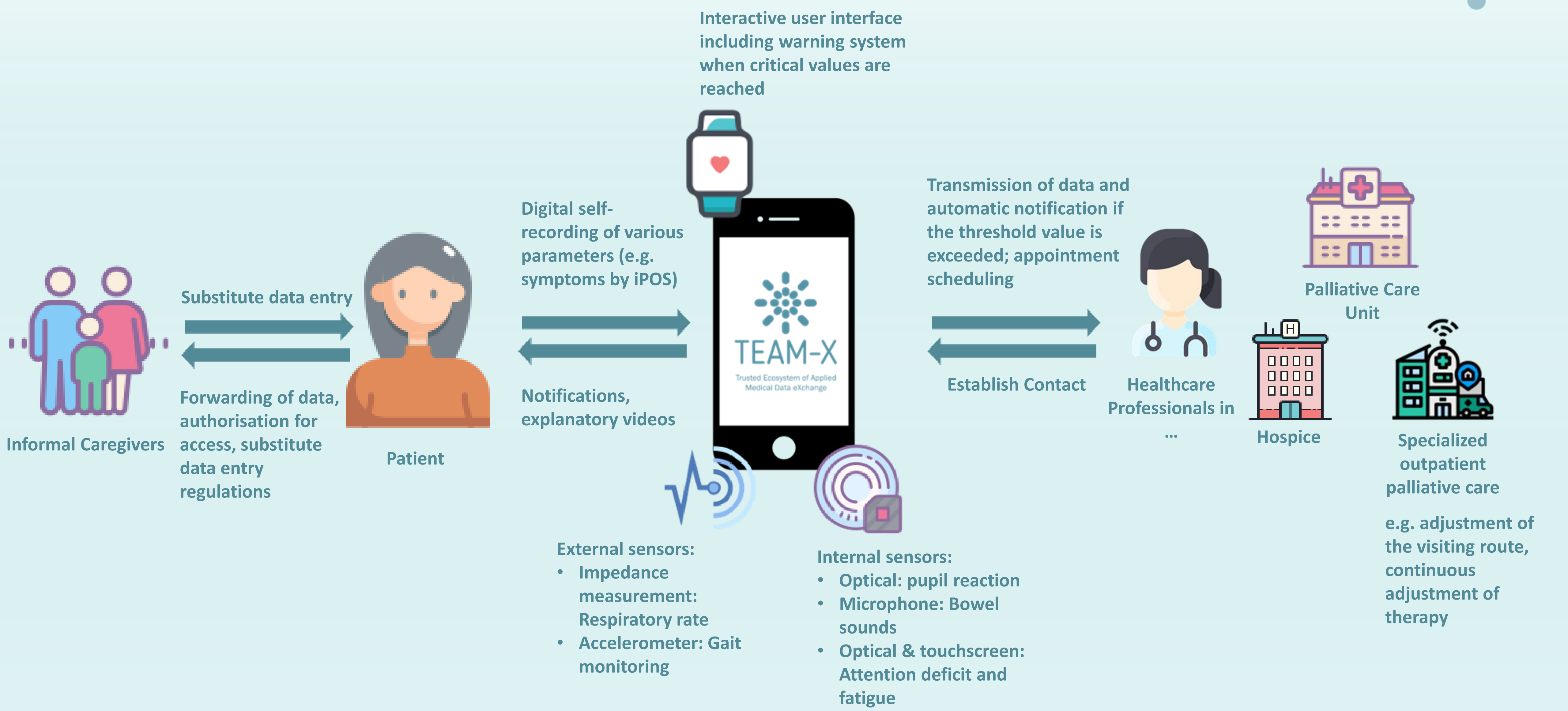

Supplement: Supplementary file 1 — Supplementary Material 1: Shortened presentation of the TEAM-X system [file 12904_2026_2020_MOESM1_ESM.pdf]
