# Supplementary material for: New technology must support, not restrict humaneness: a qualitative interview study on the potential influences of a new digital system on specialist palliative home care
Source: BMC Palliat Care. 2026 Mar 4;25:87. doi: 10.1186/s12904-026-02020-4 (PMC13063643; doi:10.1186/s12904-026-02020-4)
Supplement: Supplementary file 2 — Supplementary Material 2: Shortened interview guide [file 12904_2026_2020_MOESM2_ESM.pdf]

Interview guideline
Semi-structured interviews with healthcare professionals and relatives in specialized palliative outpatient care in the TEAM-X project

- **Welcome**
- **Brief introduction of GAIA-X and TEAM-X**
- **Introduction**

1. **Narrative impulse: How is data currently recorded and passed on in everyday specialized palliative outpatient care?**
   1.1 How are/ were the symptoms, well-being and changes in health status of palliative care patients currently recorded?

2. **Narrative Impulse: Imagine the system was installed tomorrow - what would change?**
   (preceded by a brief introduction to the possible applications of TEAM-X)
   2.1 How would the implementation of the TEAM-X data ecosystem change your and your patient's everyday care?

3. **Narrative Impulse: What is your assessment of the system?**
   - ➔ Does the approach presented make sense from your point of view? Or what would it take for you to perceive it as useful?
   - ➔ Under what conditions would it make sense to you?
   - ➔ What would you wish for?
   - ➔ Under what conditions would you use/not use the system?
   - ➔ What are the limitations of monitoring symptoms and well-being and monitoring changes in general condition using the TEAM-X data ecosystem?
   - ➔ What concerns do you have?

4. **Narrative Impulse: What do you think is needed to ensure that the TEAM-X data ecosystem can be used and integrated well into healthcare in the long term?**
   - ➔ What skills are needed? For patients, relatives and caregivers?
   - ➔ Do they need specific training?
   - ➔ Are there any requirements from an organizational perspective?
   - ➔ What must be taken into account when developing the system?
   - ➔ What expectations and requirements do you have of the design?

- **Summary**
- **Thanks and Farewell**
